# Enhancing Communication: Advancing Inpatient Care in a Tertiary Psychiatric Hospital

**DOI:** 10.1192/j.eurpsy.2026.11679

**Published:** 2026-07-31

**Authors:** S. Y. L. Lee

**Affiliations:** Institute of Mental Health, Singapore, Singapore

## Abstract

**Introduction:**

The 34th European Congress of Psychiatry in 2026 emphasises improving care and expanding horizons through enhanced collaboration with multidisciplinary professionals, experts by experience, and carers. This focus provides an opportune context to share insights from Singapore’s experience in addressing communication challenges within mental health inpatient services. Patient’s experience surveys consistently identify communication gaps as a primary concern in psychiatric inpatient settings, with interaction and communication problems, inadequate understanding of care plans, and insufficient follow-up communication emerging as predominant issues.

**Objectives:**

A standardised inpatient communication process, which enhances multidisciplinary team’s (MDT) coordination and improves family engagement throughout the patient journey, was developed and implemented in the Institute of Mental Health. It is a comprehensive communication framework mapping four key touchpoints: admission orientation within 24 hours, initial clinical updates between 24-72 hours post-admission, ongoing interim updates during admission, and discharge planning communication. The process integrates with existing Care Coordination Model activities, establishing clear roles for clinicians, advanced practice nurses, staff nurses, care coordinators and case managers. Standardised message templates were created for various communication scenarios, utilising multiple channels including SMS, WhatsApp messages, and telephone contact.

**Methods:**

The initiative was piloted across eight acute regional wards with planned adaptation for subspecialty units across the hospital. Change management strategies included pre-rollout engagement sessions with stakeholders, educational materials, publicity roadshows and post-rollout troubleshooting and feedback collection.

**Results:**

The standardised communication process aims to improve patient and family experience scores, reduce communication-related complaints, and enhance care coordination. Success will be measured through internal Patient-Reported Experience Measures and Ministry of Health’s Patient Experience Survey results, with particular focus on improving team-based care and communication metrics.

**Conclusions:**

This systematic approach addresses critical gaps in mental health inpatient communication, potentially serving as a model for other psychiatric institutions seeking to enhance family engagement and patient experience. The integration of structured communication protocols with existing care coordination frameworks represents an innovative approach to enhancing family engagement and patient experience in mental health settings.

**Disclosure of Interest:**

None Declared

